# Description of *Longidorus cheni* sp. n. (Nematoda, Longidoridae) from China

**DOI:** 10.3897/zookeys.744.23265

**Published:** 2018-03-19

**Authors:** Eda Marie Barsalote, Hoa Thi Pham, Stela Lazarova, Vlada Peneva, Jingwu Zheng

**Affiliations:** 1 Laboratory of Plant Nematology, Institute of Biotechnology, College of Agriculture & Biotechnology, Zhejiang University, Hangzhou 310058, China; 2 Institute of Biodiversity and Ecosystem Research, Bulgarian Academy of Sciences, 2 Gagarin Street, 1113 Sofia, Bulgaria; 3 Ministry of Agriculture Key Lab of Molecular Biology of Crop Pathogens and Insects, Hangzhou 310058, China

**Keywords:** D2–D3 region of large subunit (LSU) 28S rDNA, morphology, phylogeny, small subunit (SSU) 18S rDNA, taxonomy

## Abstract

*Longidorus cheni* **sp. n**., an amphimictic species recovered from the rhizosphere of *Larix principis-rupprechtii* and *Pyracantha fortuneana* in Shanxi and Beijing, China, is described and illustrated. The taxonomic position of *L. cheni* **sp. n.** among other species within the genus was elucidated using morphometric and molecular data, and phylogenetic relationships were inferred using D2–D3 expansion domains of 28S and 18S rRNA genes by Bayesian Inference (BI) method. The new species is characterised by females with a medium body size (L = 4.9–6.6 mm), a lip region slightly expanded, broadly rounded frontally and laterally, the amphidial fovea broad and symmetrically bilobed at base, odontostyle long and slender (143–168 μm), odonthophore slightly swollen at the base, tail short bluntly conoid to rounded. Guide ring located far posterior from the oral aperture (70–93 μm). Males with two ad-cloacal pairs of supplements preceded by a row of 10–14 ventromedian supplements, with robust spicules measuring 111–126 μm along the median line. Three juvenile stages were present, tail shape of J1 elongate conoid while in J2 and J3 the tail gradually becomes bluntly rounded. Codes for identifying the new species are: A6-B3-C5-D2-E2-F3-G1-H1-I2-J2-K2. *Longidorus cheni* **sp. n.** belongs to a group of species with a guide ring at the mid-odontostyle position that have a predominantly Asiatic origin. It differs from all of them by a combination of morphological characters and unique sequences of partial 18S and D2–D3 region of 28S rRNA genes. The percentage dissimilarities in partial 18S and D2–D3 28S rRNA genes of *L. cheni* to the closest species (*L. litchii*, *L. fangi*, *L. jonesi* and *L. juglans*) were 1.5 %–1.8 % and 16.8–18.3 %, respectively.

## Introduction

Longidorids, despite their long history of research (the first species of the family *Longidorus elongatus* (de Man, 1876) was described almost one hundred and fifty years ago) continue to attract the attention of scientists due to their high species diversity, wide distribution, and economic importance. The valid *Longidorus* Micoletzky, 1922 species described to date reached 167 (Peneva et al. 2013, Xu et al. 2017) with the proposed synonymy of two species (Sturhan 2014, Tanha Maafi et al. 2015). Present records of *Longidorus* distribution in mainland China reported by Guo et al. (2011) and Xu et al. (2017) include 16 species, half of which were originally described from the country: *L. jiangsuensis* Xu & Hooper, 1990, *L. fangi* Xu & Cheng 1991, *L. henanus* Xu & Cheng 1992, *L. litchii* Xu & Cheng 1992, *L. hangzhouensis* Zheng, Peng, Robbins & Brown, 2001, *L. camelliae* Zheng, Peneva & Brown, 2000, *L. asiaticus* Triscuizzi, Archidona-Yuste, Troccoli, Fanelli, Luca, Vovlas & Castillo, 2015 and *L. juglans* Xu, Ye, Wang, Zeng & Zhao, 2017.

In a survey during August 2014 and May 2015, a new species of *Longidorus* was recovered from native conifers growing in a mountainous region of Shanxi and evergreen shrubs growing in a botanic garden in Beijing, the localities situated in northern and northeastern China, respectively. Molecular approaches and phylogenetic studies in combination with morphometric characters are used as a taxonomic standard for species identification and delimitation (Gutiérrez-Gutiérrez et al. 2013, Peneva et al. 2013, Archidona-Yuste et al. 2016). The study aims to characterise this undescribed nematode species based on morphological characters coupled with molecular data and infer the phylogenetic relationships with the other species of genus *Longidorus*.

## Materials and methods

### Nematode sampling

Specimens examined in this study were extracted from soil samples collected from the rhizosphere of *Larix principis-rupprechtii* Mayr. from Shanxi and *Pyracantha fortuneana* (Maxim.) from Beijing, China. Five hundred grams (500 g) of soil were mixed and washed using a decanting and sieving technique (Brown and Boag 1988). The extract was left for two days on a Baermann funnel and the suspension was collected afterwards. Collected nematodes were examined under a stereomicroscope and *Longidorus* specimens were picked out and transferred to Syracuse dishes for storage. For morphometric studies the nematodes were killed, fixed with hot formalin, and processed to glycerine (Seinhorst 1959) as modified by De Grisse (1969). The micrographs, measurements, and drawings of nematodes were completed with the help of Nikon eclipse Ni-U 931845 compound microscope. All measurements were presented in micrometres (µm) and expressed as a mean ± standard deviation.

### DNA Extraction, amplifications, and sequencing

DNA was extracted from a single adult nematode, carefully handpicked from nematode suspensions, transferred onto a glass slide containing a 13 µl H_2_O, and cut into two pieces using a sterilised scalpel. The nematode fragments were pipetted up to 10 µl and transferred to Eppendorf tubes with 8 µl Mg+ free buffer and 2 µl proteinase K (Ye et al. 2004). PCR tubes were centrifuged at 12000 rpm for 2 minutes and immediately frozen at -70 °C for at least 30 minutes. Subsequently, each tube was incubated for 65 °C for 3 hours and nematode was digested at 75 °C for 60 minutes and 95 °C for 10 minutes. Finally, the DNA suspensions were cooled down at 8 °C and stored at -20 °C until use. A total of 25 µl PCR mixture was prepared containing 2.5 µl LA buffer, 2 µl dNTP, 1.5 µl each primer (synthesized by Takara Company, Shanghai, China) and 3 µl DNA template, 0.3 µl LATaq and 14.2 µl distilled water. All PCR reactions were conducted in the S1000 thermal cycler (BIO-RAD). Fragments of 18S and 28S region were amplified using two sets of primers: forward primer SSU_F_07 (5’ AAA GAT TAA GCC ATG CAT G 3’) and reverse primer SSU_R_81 (5’ TGA TCC ACC TGC AGG TTC AC 3’) (Gutiérrez-Gutiérrez et al. 2011) and forward primer D2A (5’ ACA AGT ACC GTG AGG GAA AGT TG 3’) and reverse primer D3B (5’-TCG GAA GGA ACC AGC TAC TA-3’) (De Ley et al. 1999), respectively. The thermal cycling protocol consisted of denaturation at 95 °C for 5 minutes, followed by 35 cycles of denaturation at 94 °C for 30 seconds, annealing at 55 °C for 45 seconds, extension at 72 °C for 2 minutes and a final extension at 72 °C for 10 minutes. After DNA amplification, 2.5 µl aliquots of PCR products were analysed by gel electrophoresis in 1 % agarose gel (100V, 400 mA, 30 minutes) stained in ethidium bromide for 10 minutes and DNA were visualized under UV illumination. Amplified PCR products were purified following the instructions as described in the nucleic acid purification kit of AXYGEN (catalogue No. AP-GX-250) of the AXYGEN Biotechnology Co., Ltd. Hangzhou, China. Purified DNA were ligated to pUCM-T vector and transformed in to DH 5alpha competent cells. The transformants were screened on an ampicillin agar LB plates containing 400 ml IPTG, X-Gal and left at 37 °C overnight. White colonies were selected, transferred to 5 ml LB containing 100 mg/ml ampicillin, and incubated at 37 °C for 16–24 hours. PCR amplification was further confirmed with the primer insertion and expected band; four clones were sequenced per population. Sequencing was done at the SANGON Biotechnology Co., Ltd. Since the clones were identical, only one sequence for each gene has been deposited in GenBank sequence database with the following accession numbers: KY284157 and KF270638 for D2–D3 expansion domains of 28S rDNA, KF261570 and MG656980 for the 18S rDNA region.

### Sequence and phylogenetic analyses

The D2–D3 28S and 18S rDNA sequences were compared with those of other nematode species deposited in GenBank database using BLASTn similarity search tool. The homologous sequences nearest to those of the new species were aligned using the GUIDANCE2 Server available at http://guidance.tau.ac.il/ with default parameters (Sela et al. 2015) and manually trimmed and edited in Mega 7 (Kumar et al. 2016). Bayesian Inference (BI) algorithm implemented in MrBayes 3.2.5 was used for phylogenetic relationships reconstructions (Huelsenbeck and Ronquist 2001, Ronquist et al. 2012). For further details, see Lazarova et al. (2016). The 50 % majority rule consensus BI trees of *Longidorus* and *Paralongidorus* spp. are based on a multiple sequence alignment data sets that included: a) 57 sequences and 700 total characters for D2–D3 28S rRNA gene and b) 48 sequences and 993 total characters for 18S rRNA gene.

## Taxonomy

### Longidorus cheni sp. n.

Taxon classificationAnimaliaNematodaLongidoridae

http://zoobank.org/AD7993D5-AB3D-4436-863D-2D469EEE49CA

**Figure 1.**
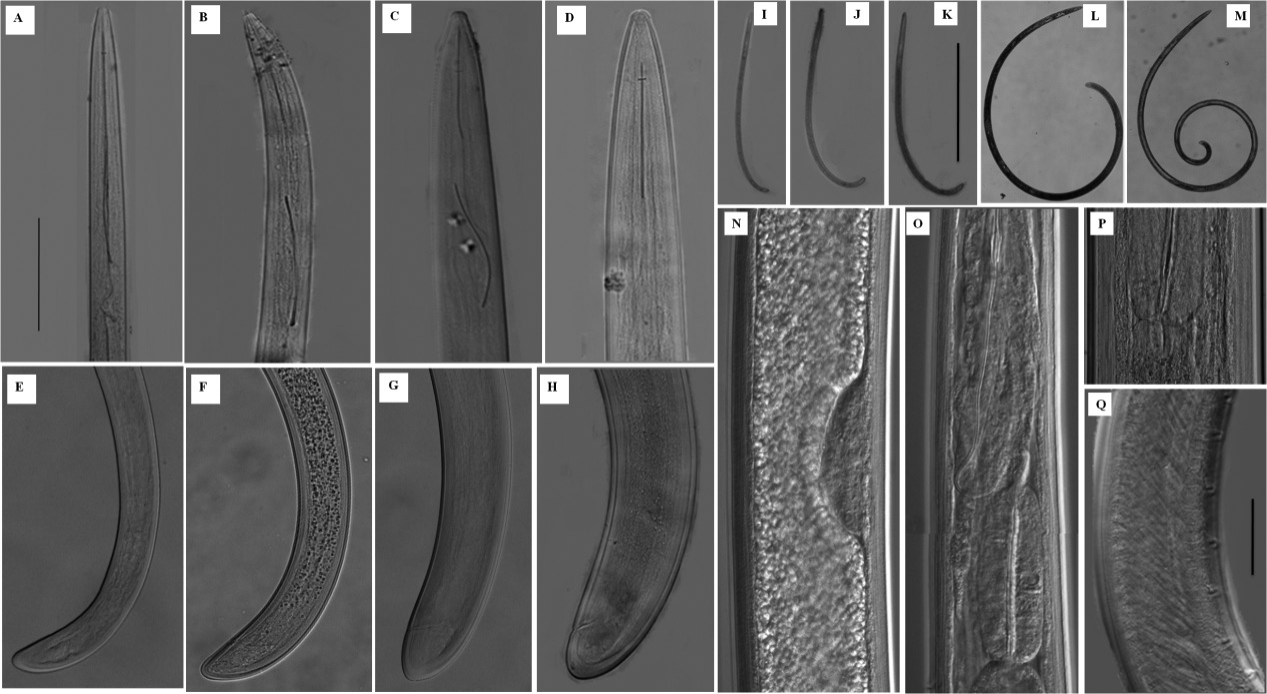
*Longidorus cheni* sp. n. Juveniles: **A–C** Anterior region of first-, second- and third-stage **E–G** Posterior end of first-, second- and third-stage **I–K** Habitus of first-, second- and third-stage juvenile **N** developing gonad in a second stage juvenile. *Female*: **D** Anterior end **H** Tail **L** Habitus **O** Pharyngeal bulb region **P** Cardia *Male*: **M** Habitus **Q** Ventromedian supplements. Scale-bars: 60 μm (**A–H**); 100 µm (**I–M**); 15 µm (**N–Q**).

**Figure 2.**
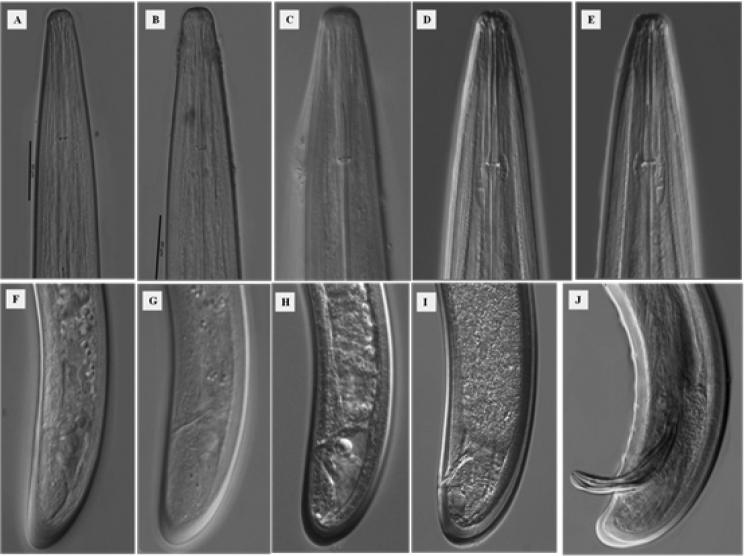
*Longidorus cheni* sp. n. Juveniles: **A–C** Head region of first-, second- and third-stage **F–H** Head region of first-, second- and third-stage. *Female*: **D** Head end **I** Tail end; *Male*: **E** Head end **J** Tail end. Scale bar: 20 µm (**A–J**).

**Figure 3.**
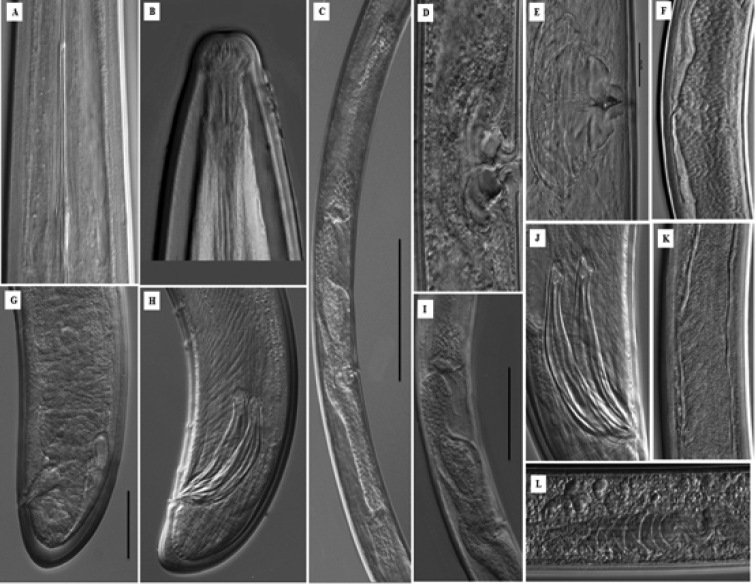
*Longidorus cheni* sp. n. Female: **A** Odontophore region **B** Amphidial fovea shape **C** Reproductive system **D–E** Vulval region **F** Sperms inside uteri **I** Oviduct separated by sphincter **G** Tail region **L** Ovary. *Male*: **H** Tail end **J** Spicule **K** Testis with sperms. Scale bars: 20 µm (**A, B, E, G, H, J**); 100 µm (**C**); 50 µm (**D, F, I, K, L**).

**Figure 4.**
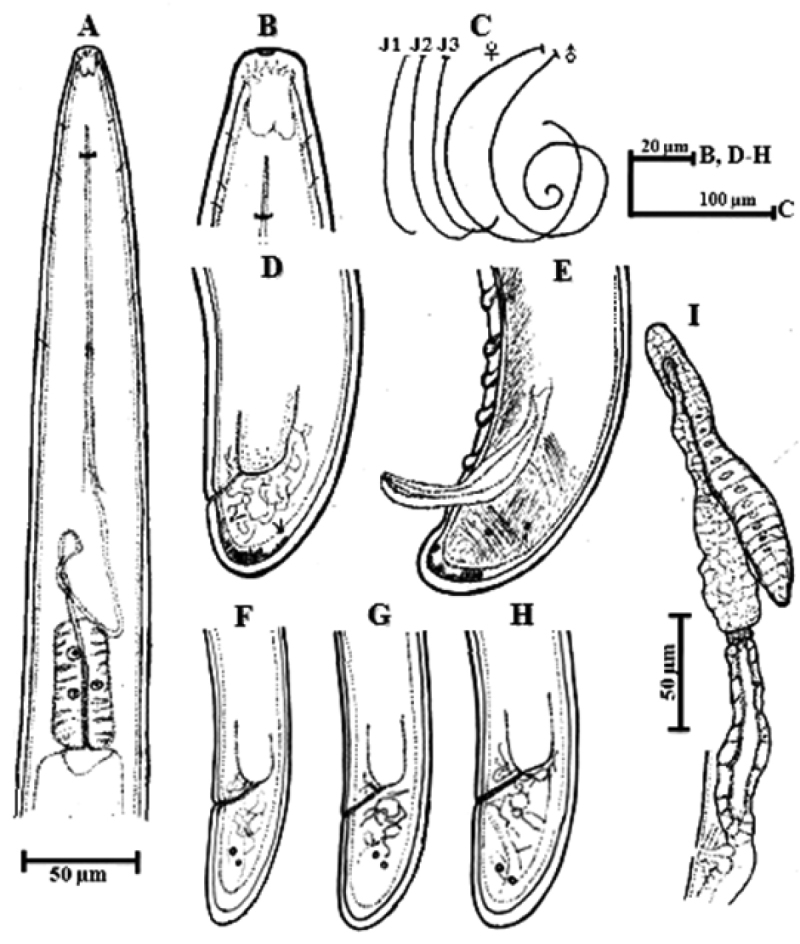
*Longidorus cheni* sp. n. Female: **A** Anterior region **B** Amphidial fovea shape **C** habitus **D** Tail region **I** Anterior genital branch. *Male*: **C** Habitus **E** Tail end. *Juveniles*: **C** Habitus **F–H** Posterior end of first-, second- and third- stage.

**Figure 5.**
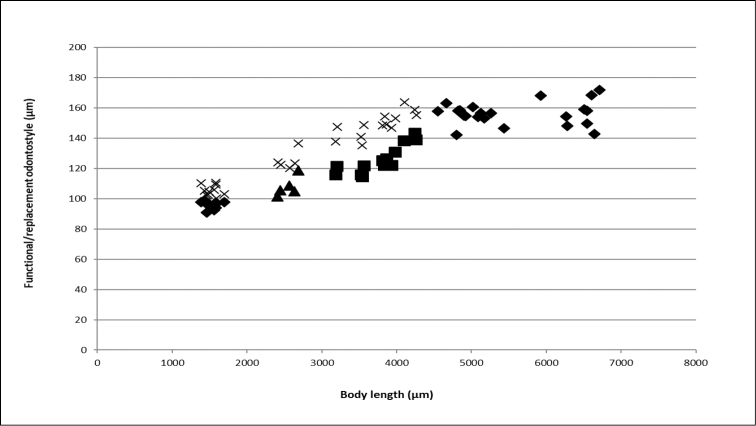
Scatter plot of odontostyle (◆, ▲, ◼) and replacement odontostyle (×) against body length of *Longidorus cheni* sp. n. juveniles (J1 to J3) and (◆) adults.

Figures 1 , 2 , 3 , 4 , 5

#### Material examined.

Twelve females, twelve males, fifty-two juveniles (J1-J3) from Shanxi province and ten females, four males, thirty juveniles (J1-J3) from Beijing.

#### Description.

*Measurements* (see Tables 1 and 2).

**Table 1.** Measurements (in µm and in the form, mean ± standard deviation and range) of females and males of *Longidorus cheni* sp. n. from two provinces in China.

Origin	Holotype	Shanxi		Beijing	
		Paratypes		Paratypes	
Host	Female	*Larix principis-rupprechtii*		*Pyracantha fortuneana*	
		Females	Males	Females	Males
N		12	12	10	4
L	6606	5778.1 ± 740.7 (4924–6645)	5334.7 ± 731.05 (4553–6709)	5675 ± 687.2 (4125–5678)	5109±686.4 (4153–6548)
a	63.1	51.98 ± 4.6 (47.8–63.1)	61.4 ± 5.6 (52.7–69.1)	49.8 ± 4.1 (45.7–59.0)	58.7±7.9 (46.2–69.0)
b	10.2	9.68 ± 1.79 (7.5–12.4)	9.1 ± 1.5 (7.4–12.2)	9.2 ± 1.79 (7.5–12.2)	8.7±1.3 (7.1–11.4)
c	153.3	133.13 ± 15.04 (115.9–153.3)	108.53 ± 6.56 (100.8–120.1)	135 ± 14.4 (118.0–149.0)	103.1±10.4 (86.1–120.1)
c’	0.68	0.74 ± 0.08 (0.63–0.86)	0.8 ± 0.09 (0.64–0.97)	0.78 ± 0.09 (0.62–0.86)	0.81 ± 0.1 (0.68–0.99)
V	43.2	44.07 ± 3.39 (40.6–49.4)	–	46.4 ± 2.89 (40–48.3)	–
Odontostyle	168	155.7 ± 6.6 (143–168)	156.8 ± 8.85 (142–172)	153.2 ± 5.03 (142–166)	156.3 ± 9.3 (142–173)
Odontophore	103	90.5 ± 7.0 (81.5–103)	88.8 ± 4.9 (8.6–99)	90.0± 6.04 (82–102)	83.3 ± 5.1 (73–86)
Guide ring to anterior end	85	77.6 ± 5.9 (70–91)	78.5 ± 3.4 (74–85)	78.5±3.6 (74–84)	79.3 ± 6.7 (72–93)
Lip width	20	19.7 ± 1.2 (18–22)	19.6 ± 1.2 (17–21)	18.6 ± 2.2 (17.5–23)	19.7 ± 1.3 (17–21)
Width at guide ring	55	49.0 ± 5.2 (42–57)	46.9 ± 4.7 (40–55)	46 ± 4.2 (42–55)	46.5 ± 4.5 (39–55)
Width at anus	63	60.3 ± 7.6 (49–72)	61.9 ± 5.7 (54.5–70)	60 ± 7.4 (48–70)	61.2 ± 5.5 (55–70)
Tail length	33	26.4 ± 7.8 (24–33)	29.1 ±3.1 (27.6–32.2)	26.1±7.7 (25–34)	29.8 ± 4.2 (28–34)
Spicule	112	–	112.3 ± 7.8 (101–124)	–	111.3 ± 7.2 (101–121)

**Table 2.** Measurements (in µm and in the form, mean ± standard deviation and range) of juvenile stages of *Longidorus cheni* sp. n. from two provinces in China.

Origin	Shanxi			Beijing	
	Paratypes			Paratypes	
Stages	J1	J2	J3	J2	J3
N	17	15	20	12	18
L	1582 ± 150. 6 (1390–1929)	2822 ± 390.3 (2413–3539)	3711.5 ± 380.3 (3205–4269.5)	2489 ± 132.5 (2375–3529)	3787.5 ±298.3 (3339–4909)
a	39.8 ± 3.6 (34.6–48.6)	50.54 ± 5.7 (42.9–62.7)	54.3 ± 7.0 (43.3–69.7)	47.7 ± 2.4 (44–59)	57 ± 4.71 (43–67)
b	4.6 ± 0.5 (4.0–5.8)	6.7 ± 1.1 (5.1–8.9)	7.9 ± 1.5 (6.2–11.3)	6.2 ± 0.8 (5.9–8.5)	7.9 ± 2.1 (5.9–10.6)
c	44.2 ± 4.6 (38.2–54.0)	69.03 ± 10.2 (54.8–89.4)	84.9 ± 8.1 (74.45–103.3)	63.9 ± 4.2 (48.0–75.0)	78.3 ± 9.2 (65.1–103.0)
c’	1.25 ± 0.12 (1.02–1.46)	0.99 ± 0.16 (0.75–1.33)	0.82 ± 0.09 (0.68–1.02)	0.9 ± 0.12 (0.8–1.5)	0.81 ± 0.1 (0.65–1.25)
Total stylet	148.9 ± 10.4 (137–174)	180.5 ± 12.0 (165–200.5)	220.8 ± 13.0 (198–246)	178.9± 10.7 (167–195)	212.0 ± 10.2 (193–247)
Odontostyle	96.8 ± 4.2 (91–109)	109.8 ± 6.4 (100–118.5)	133.2 ± 11.7 (121–157)	107 ± 3.3 (101–116)	131.0 ± 5.2 (120–146)
Odontophore	52.2 ± 7.0 (44–66)	70.7 ± 7.2 (64–85)	87.6 ± 3.3 (83–94)	71.9 ± 6.7 (65–89)	81.0 ± 8.1 (83–89)
Replacement odontostyle	103.9 ± 4.3 (96–110.5)	130.9 ± 5.3 (125–141)	150.63 ± 6.48 (143–164)	132 ± 3.4 (129–146)	152.0 ± 4.35 (146–160)
Guide ring to anterior end	43.1 ± 4.0 (39–55)	55.5 ± 6.6 (44.5–65)	65.8 ± 6.1 (57–78)	52.9 ± 3.9 (47–69)	64.0 ± 5.25 (53–75)
Lip width	11.1 ± 0.8 (9–12)	14.6 ± 1.9 (11–18)	15.6 ± 1.8 (13–19)	13.9 ± 0.67 (10–18)	15.0 ± 1.1 (12–19)
Body width at guide ring	26.3 ± 2.9 (22.5–33)	34.1 ± 6.4 (25–46.5)	42.8 ± 9.6 (33–67)	26.3 ± 2.9 (22.5–33)	44.08 ± 6.39 (35–67)
Anal body width	28.9 ± 3.9 (22–38)	42.4 ± 5.4 (33–52)	54.3 ± 8.09 (45–68)	43.2 ± 3.9 (43–56)	52.4 ± 5.4 (43–72)
Tail length	35.9 ± 3.1 (31- 39)	33.17 ± 1.3 (28–34)	32.3 ± 3.3 (30–35.5)	33.8 ± 0.9 (29–35)	31.87 ± 3.3 (29–35)

*Female.* Body habitus G-shaped when relaxed by gentle heat (Fig. 1M) gradually tapering in both ends. Cuticle under light microscope with three distinct layers, the middle one consisting of several sub-layers, slightly refractive, most pronounced at labial and tail regions, the inner one thicker at labial and tail region with radial striations, cuticle 6 μm thick at post-labial area, 5 μm along the body and 11 μm in post-anal region. Nine lateral, three dorsal and five ventral body pores in the neck region. Lip region slightly expanded, broadly rounded frontally and laterally (Fig. 2D). Amphidial fovea broad and symmetrically bilobed at the base (Fig. 3B). Odontostyle long and slender with simple base, odontophore slightly swollen at the base (Fig. 3A), approx. 1/3 of the odontostyle length. Guide ring located far posterior from oral aperture (Fig. 4A–B). Pharynx dorylaimoid with anterior part more or less coiled, pharyngeal bulb comparatively short measuring 107–138 × 23–28 μm (Fig. 1O). Arrangement of pharyngeal glands normal, dorsal gland nucleus located at 23–34 μm and ventrosublateral gland nuclei at 48–54 μm from the beginning of pharyngeal bulb. Pharyngo-intestinal valve (cardia) hemispherical (Fig. 1P). Tail short dorsally convex and terminus bluntly conoid with two pairs of caudal pores (Fig. 2I). Vulva a transverse slit located slightly anterior from mid body (V = 40–48 %), vagina well developed extending nearly half of body diameter (Fig. 3D, E). Reproductive system amphidelphic with anterior and posterior branches almost equally developed (Fig. 3C). Sperms observed in the uteri of most females (Fig. 3F).

*Male.* Morphologically similar to female. Body G to spiral shape (Fig. 1M). Testes paired and fully developed. Sperms abundant and irregularly shaped (Fig. 3K). Spicules robust (Fig. 3J). Lateral guiding piece 32–35 μm. Two ad-cloacal pairs of supplements preceded by row of 10–14 ventromedian supplements (Fig. 3H). Tail ventrally curved bluntly conoid to hemispherical, 2–3 lateral pores on each side (Fig. 3H).

*Juveniles.* Three juvenile stages (J1-J3) distinctly separated by differences in the body length, odontostyle and replacement odontostyle length (Fig. 1A–C). In the first stage juvenile, the anterior part of replacement odontostyle is inserted in the wall of odontophore (Fig. 3A). Morphologically, juveniles resemble adults except for the smaller size and not developed reproductive system. Habitus assuming J shape does not change with the stage (Fig. 1I–K). Tail length does not change while anal width increases (Fig. 1E-G) thus **c**’ ratio decreases (Table 2), guide ring position becoming more posterior during successive stages (Fig. 2A–C). First stage juvenile is characterized by a conoid tail becoming bluntly conoid in second to third stages (Fig. 2F–H).

#### Sequences and phylogenetic analyses.

**Figure 6.**
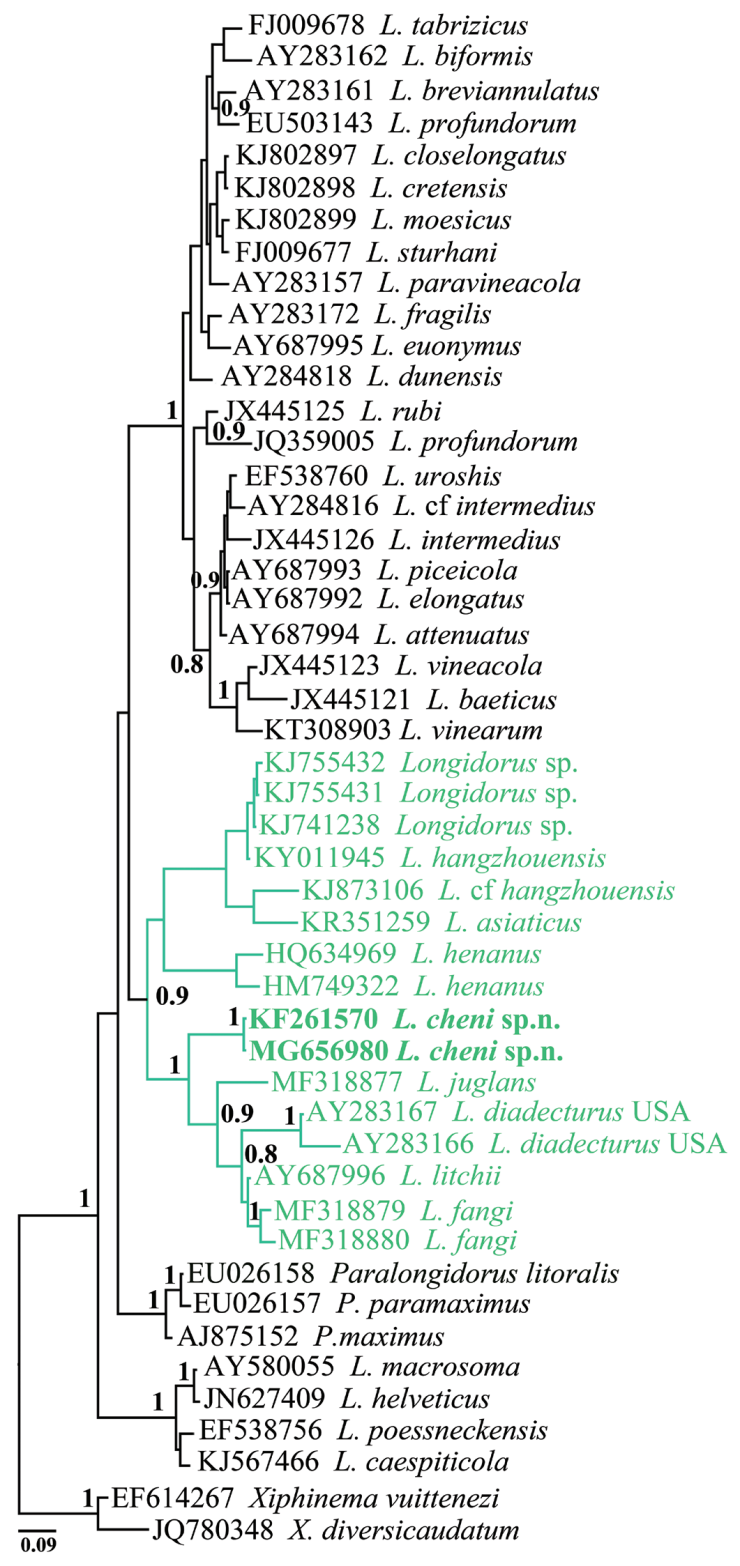
Phylogenetic tree using 18S rDNA and inferred from a Bayesian analysis with GTR+G model and *Xiphinema* spp. as an outgroup. Posterior probabilities ≥ than 0.8 are presented.

**Figure 7.**
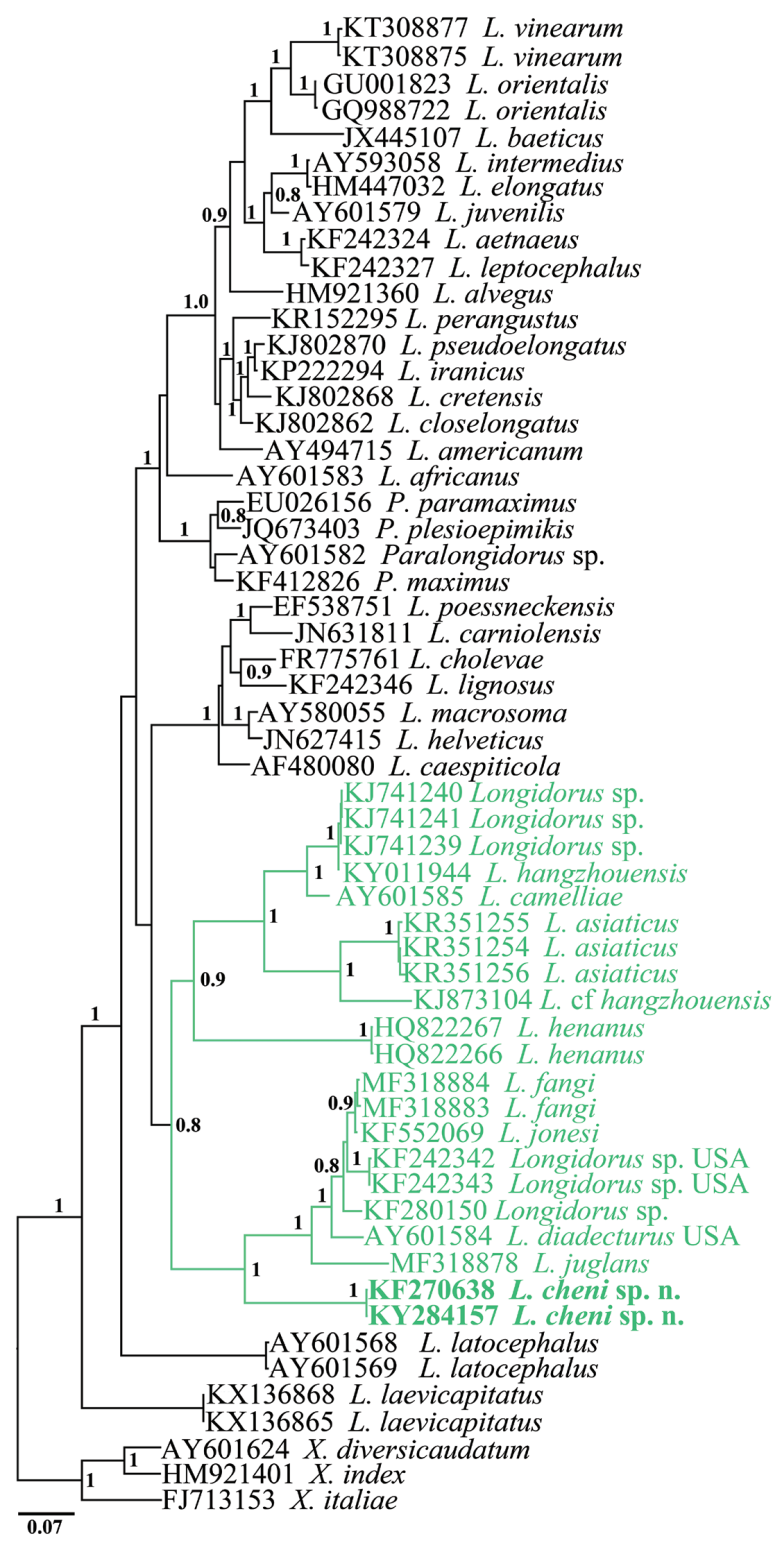
Phylogenetic tree using D2–D3 28S rDNA and inferred from a Bayesian analysis with GTR+G model and *Xiphinema* spp. as an outgroup. Posterior probabilities ≥ than 0.8 are presented.

The length of PCR products based on gel images of the amplification of partial 18S and D2–D3 region of 28S RNA genes of *L. cheni* sp. n. (LDT235 and BJ07) was 844 bps and 856 bps, respectively. The sequences of both populations were identical. The phylogenetic relationships of *L. cheni* sp. n. with the closest species inferred from analyses of the partial 18S rDNA and D2–D3 expansion segments of 28S rDNA sequences using BI are presented in Figs 6 and 7, respectively. In general, the new species grouped with other *Longidorus* species of predominantly Asiatic origin in both phylogenetic reconstructions. In D2–D3 rDNA phylogenetic tree, *L. cheni* clustered in a well-supported clade comprising four species from China (*L. juglans* (MF318878), *L. fangi* (MF318883-84), *Longidorus* sp. (KF280150); one from Japan (*L. jonesi* (KF552069) and two species from North America, USA (*L. diadecturus* (AY601584) and *Longidorus* sp. (KF242342-43)). With exception of the species for which there are no descriptions, all mentioned species have a guide ring at mid-odontostyle area. Similarly, in the 18S rDNA phylogenetic reconstruction *L. cheni* sp. n. clustered with the same group of species (*L. jonesi*, *L. fangi*, and *L. diadecturus*) and *L. litchii* (AY687996) that has no D2–D3 rDNA sequence deposited in GenBank. The percentage dissimilarities of *L. cheni* to the closest species *L. litchii*, *L. fangi*, and *L. juglans* in 18S rRNA gene were 1.5 %, 1.8 %, and 1.8 %, respectively (a total of 955 positions in the final dataset). Much higher were the pairwise percentage distances of *L. cheni* sp. n. to the closest species in D2–D3 28S rRNA gene ranging from 16.8–16.9 % (*L. fangi* and *L. jonesi*) to 18.3 % (*L. juglans*).

#### Type habitat and locality.

Specimens were recovered from soil around the roots of a conifer (*L. principis-rupprechtii*) and Chinese firethorn (*P. fortuneana*) in mountainous region of Shanxi and botanic garden in Beijing, China, GPS coordinates 37°50'815"N, 111°27'253"E and 30°34'54.7"N, 114°15'40.9"E, respectively.

#### Type material.

Holotype. Female slide no. LS5313 and paratypes (slides no. LS 5301–5312, LS 5314–5350) includes 12 females, 12 males and 52 juveniles deposited in the Nematode collection C602 Nematology laboratory of Zhejiang University, Hangzhou, China. One female, one male, three juveniles deposited at the nematode collection of the Institute of Biodiversity and Ecosystem Research, Bulgarian Academy of Sciences, Sofia, Bulgaria.

#### Etymology.

The species is named after Prof. Pinsan Chen, Institute of Plant Protection, Chinese Academy of Agricultural Sciences, one of the pioneer plant nematologists in China.

#### Diagnosis and relationship.

*Longidorus cheni* sp. n. is an amphimictic species characterized by females with medium body size (L = 4.1–6.6 mm), assuming G-shape, lip region 16–23 μm wide, posteriorly situated guide ring (at 70–93 μm from anterior end), long odontostyle (142–168 μm), odontophore base slightly swollen, tail short (24–33 μm) and bluntly conoid to rounded. Males abundant, spicules 111–123 μm long, ventromedian supplements 10–14. Three juvenile stages present. The tail of the first stage juvenile conoid, tail shape in the second and third stage juveniles gradually becoming rounded. Finally, the species have specific ribosomal sequences KY284157 and KF270638 for D2–D3 expansion domains of 28S rDNA, KF261570 and MG656980 for the 18S rDNA region. The identification codes of *L. cheni* sp. n. based on the polytomous key by Chen et al. (1997) and additional codes (Peneva et al. 2013) are: A6-B3-C5-D12-E2-F3-G1-H1-I2-J2-K2.

*Longidorus cheni* sp. n. belongs to a group of species (*L. jonesi*-group) having guide ring at mid-odontostyle area (Xu et al. 2017) which consists of *L. diadecturus* Eveleigh & Allen, 1982, *L. fursti* Heyns, Coomans, Hutsebaut & Swart, 1987, *L. himalayensis* (Khan, 1986) Xu & Hooper, 1990, *L. ishigakiensis* Hirata, 2002, *L. jagerae* Heyns & Swart, 1998, *L. jonesi* Siddiqi, 1962, *L. juglans*, *L. laricis* Hirata, 1995, *L. litchii*, *L. macromucronatus* Siddiqi, 1962, *L. martini* Merny, 1966, *L. naganensis* Hirata, 1995, *L. orongorongensis* Yeates, Van Etteger & Hooper, 1992, and *L. waikouaitii* Yeates, Boag & Brown, 1997 (See Tables 3 and 4).

**Table 3.** Partial polytomous key of *Longidorus* species with guide ring at mid-odontostyle area including *Longidorus cheni* sp. n. based on polydomous key of Chen et al. (1997), Loof and Chen (1999) and Peneva et al. (2013).

*Longidorus* species	A	B	C	D	E	F	G	H	I	J	K
***L. cheni* sp. n.**	6	3	5	12	2	3	1	1	2	2	2
*L. laricis*	7	3	5	4	2	23	2	1	2	2	7
*L. ishigakiensis*	7	2	5	1	1	3	23	12	1	2	3
*L. litchii*	567	2	5	2	2	23	12	1	2	1	7
*L. orongorongensis*	67	4	5	1	4	34	2	1	2	1	12
*L. naganensis*	6	3	5	2	2	2(3)	1	1	1	2	7
*L. fangi*	56	3	5	23	5	23	2	12	1	1	56
*L. juglans*	5	23	5	1	1	23	1	1	2	2	23
*L. jonesi*	45	2	5	1	1	2	1	1	1	2	?
*L. himalayensis*	45	2	5	2	2	2	2	1	1	?	?
*L. macromucronatus*	45	3	5	3	1	2	2	1	1	1	56
*L. waikouaitii*	4	3	5	1	4	3	12	1	1	?	?
*L. fursti*	4	23	5	4	5	2	23	12	1	1	6
*L. diadecturus*	4	23	5	2	5	2	12	1	1	?	?
*L. jagerae*	34	2	5	4	1	2	2	12	1	?	?
*L. martini*	3	12	5	4	1	2	23	12	1	?	?

Note: A – odontostyle length; B – lip region diameter; C – distance of guide ring to anterior body length; D – shape of anterior region; E – amphidial fovea shape; F – body length; G – index “**a**”; H – tail shape; I – presence/absence of male; J – number of juvenile stages; K – tail shape in first stage juvenile.

**Table 4.** Morphometric comparisons of *Longidorus cheni* sp. n. and related *Longidorus* spp. with close morphological similarities based on polytomous key for identification of species (Cheng et al. 1997).

Species	L (mm)	c’	Odontostyle length (µm)	Lip region width (µm)	Guide ring position (µm)	V
***L. cheni***	**4.12–6.64**	**0.62–0.86**	**142–173**	**16–23**	**70–94**	**40–49.4**
*L. laricis*	4.65–5.97	0.64–0.9	160–183	16–18	84–100.5	45.8–51.2
*L. ishigakiensis*	5.31–6.85	1.0–1.2	158–181	13–14	83–95	45.4–51
*L. litchii*	4.14–5.29	0.61–0.79	138–171	12.5–14	82.5–96.5	49–54
*L. orongorongensis*	6.03–7.99	0.61–0.73	152–166	22–23	63–73	49–54
*L. naganensis*	3.83–5.18	0.69–0.89	141–160	16–18	77–89	47.1–54.3
*L. fangi*	4.6–5.52	0.75–1.12	124–144	16–18	69.5–87	48–55
*L. juglans*	3.90–5.25	0.6–0.9	125–140	14–18	69–78	47.1–50.7
*L. macromucronatus*	4–4.9	0.63–0.8	117–128	14*	58–68	43–47.8
*L. himalayensis*	3.42–3.9	0.7–0.8	115–125	15	55–60	47.4–50.1
*L diadecturus*	3.32–4.02	0.77–0.94	109–121	15–16	50–64	44–48
*L. jonesi*	3.17–3.8	0.6–0.87	107–120	23*	57–66	50.0–52.4
*L. waikouaitii*	6.44–7.17	0.51–0.74	113–117	16.5–17	56.5–59.5	48.6–53.1
*L. jagerae*	3.10–3.87	0.8–1.02	95–109	11.5–12.5	62–81	51.5–56.3
*L. fursti*	3.93–5.08	0.9–1.14	99.5–108	14.5–16	64–73	51.5–53.6
*L. martini*	2.9–4.5	1.3	83–96	11–13	51–66	52–56

*calculated from the original drawings.

*Longidorus cheni* sp. n. morphologically is most similar to *L. naganensis* from which it can be distinguished by having different first stage juvenile tail (broadly rounded *vs* digitate with mucro (**c**’ = 1.02–1.46 *vs* **c**’ = 2.0–2.5), males abundant *vs* males absent (Hirata 1995). Furthermore, it can be differentiated from all other species belonging to this group. It differs from:

*L. juglans* by females having a longer odontostyle (143–168 *vs* 107–120 μm), different amphidial fovea shape (bilobed *vs* non-bilobed) (Xu et al. 2017);

*L. laricis* by females having a smaller **a** ratio (45.7–63.1 *vs* 83–108), males abundant *vs* males rare, longer spicules (101–124 *vs* 66.2 μm), different tail shape in J1 (conoid, **c**’ = 1.02–1.46 *vs* elongate conoid with a digitate tip, **c**’ = 1.8–2.4) (Hirata 1995);

*L. litchii* by females having a smaller **a** ratio (45.7–63.1 *vs* 72–84), wider lip region (17.5–23 *vs* 12.5–14 μm), smaller **V** ratio (40–49.4 *vs* 49–54), longer spicules (101–124 *vs* 68.5–71 μm), number of ventromedian supplements (10–14 *vs* 6–7), number of stages (3 *vs* 4), different tail shape in J1 (bluntly conoid, **c**’ = 1.02–1.46 *vs* elongate conoid with a long digitate tip, **c**’ = 2.72–3.42) (Xu and Cheng 1992), odontophore base (slightly *vs* strongly flanged (Zheng et al. 2002);

*L. fangi* by females having a smaller **a** ratio (45.7–63.1 *vs* 81–98), amphidial fovea shape (bilobed *vs* non-bilobed), longer odontostyle (142–168 *vs* 124–144 μm), lower **c**’ ratio in J1 (**c**’ = 1.02–1.46 *vs* **c**’ = 1.58–2.2) (Xu and Cheng 1991);

*L. fursti* by females having a smaller **a** ratio (45.7–63.1 *vs* 105–137), wider lip region (17.5–23 *vs* 14.5–16 μm), different amphidial pouch shape (bilobed *vs* non-bilobed), longer odontostyle (142–168 *vs* 99.5–108 μm), smaller **V** ratio (40–49.4 *vs* 51.5–53.6), lower **c**’ ratio in J1 (**c**’ = 1.02–1.46 *vs* **c**’ = 2.84–2.93) (Heyns et al. 1987);

*L. himalayensis* by females having a longer (L = 4.1–6.6 mm *vs* L = 3.42–3.9) and more plump body (**a** = 45.7–63.1 *vs* **a** = 97.8–112), a wider lip region (18–23 *vs* 14–15 μm), longer odontostyle (142–168 *vs* 115–125 μm), more posteriorly situated guide ring (70–91 *vs* 55–60 μm) (Khan 1986);

*L. ishigakiensis* by females having a smaller **a** ratio (45.7–63.1 *vs* 106–130), wider lip region (18–23 *vs* 13–14 μm), different amphidial pouch shape (bilobed *vs* non-bilobed), smaller **c**’ ratio (**c**’ = 0.62–0.86 *vs* **c**’ = 1.0–1.2), different tail shape in J1 (bluntly conoid, **c**’ = 1.02–1.46 *vs* rounded, **c**’ = 1.9–2.5), males abundant *vs* males absent (Hirata 2002);

*L. jagerae* by females having a differently shaped lip region (not expanded *vs* expanded), more plump body (**a** = 45.7–63.1 *vs* **a** = 89–107), longer odontostyle (142–168 *vs* 95–109 μm), more anteriorly situated vulva (**V** = 40.0–49.4 *vs* **V** = 51.5–56.3), prerectal inclusions (absent *vs* present) (Heyns and Swart, 1998);

*L. jonesi* by females having a longer body (L = 4.1–6.6 *vs* L = 3.17–3.8 mm) and odontostyle (142–168 *vs* 107–120 μm), more posteriorly situated guide ring (70–91 *vs* 57–66 μm), more anteriorly situated vulva (**V** = 40.0–49.4 *vs* **V** = 50–52.4) (Siddiqi 1962);

*L. martini* by females having a longer body (L = 4.1–6.6 *vs* L = 3.18–4.29 mm) and odontostyle (142–168 *vs* 83–96 μm), more posteriorly situated guide ring (70–91 *vs* 51–66 μm), more anteriorly situated vulva (**V** = 40.0–49.4 *vs* **V** = 52–56) (Merny 1966);

*L. diadecturus* by females having a longer body (L = 4.1–6.6 *vs* L = 3.32–4.02 mm), odontostyle (143–168 *vs* 109–121 μm) and pharyngeal bulb (107–138 *vs* 62–83 μm), more posteriorly situated guide ring (70–91 *vs* 50–64 μm) (Eveleigh and Allen 1982);

*L. orongorongensis* by females having a shorter and more plump body (L = 4.1–6.6, **a** = 45.7–63.1 *vs* L = 6.0–8 mm, **a** = 81–106), more posterior guide ring position (70–91 *vs* 63–73 μm), smaller **V** ratio (40–49.4 *vs* 49–54), longer spicule (101–104 *vs* 84–87 μm) (Yeates et al. 1992);

*L. macromucronatus* by females having a plumper body (**a** = 45.7–63.1 *vs* **a** = 94–105), a wider lip region (17.5–23 *vs* 14 μm), longer odontostyle (142–173 *vs* 117–128 μm), 3 *vs* 4 juvenile stages, differently shaped tail in J1 (broadly conoid *vs* sub-digitate **c**’ = 1.02–1.46 *vs* **c**’ = 0.63–0.8) (Siddiqi 1962);

*L. waikouaitii* by having differently shaped amphidial fovea (pocket shaped, bilobed at the base *vs* funnel shaped), a longer odontostyle (142–173 *vs* 113–117 μm), more posterior position of the guide ring (70–91 *vs* 56.5–59.5 μm), males abundant *vs* males absent (Yeates et al. 1997).

## Discussion

Our findings on the morphology and genetics of *L. cheni* sp. n. are in agreement with the hypothesis about the common origin of *Longidorus* species having a guide ring at the mid-odontostyle area (Xu et al. 2017); furthermore, these species have the odontophore base slightly or strongly flanged (with exception of the species from New Zealand) and bluntly rounded to a hemispherical tail (code H1(2)). More than half of the species of this group with known juveniles develops through three stages (the only exception is *L. litchii*), all of them occurring in South East Asia (Table 4). This shows a characteristic biogeographical pattern of *Longidorus jonesi*-group being spread in South East Asia (China and Japan, eight species), North India (three), New Zealand (two), South Africa (SA, Rhodesia, three), North America (Canada, USA, two), and only one species (*L. jonesi*) reported from two regions (India, Japan). The highest number of species in South East Asia suggests this region as a probable centre of origin of *Longidorus jonesi*-group.

## Supplementary Material

XML Treatment for Longidorus cheni
